# Eosinophil Granule Proteins in Chronic Rhinosinusitis: A Systematic Review of Biomarker Utility for Disease Characterization and Outcomes

**DOI:** 10.3390/jcm15207790

**Published:** 2026-10-09

**Authors:** Saima N. Farook, Joshua B. Smith, Jacquelyn K. Callander

**Affiliations:** 1 Department of Otolaryngology-Head and Neck Surgery, Saint Louis University School of Medicine, St. Louis, MO 63104, USA; 2 Department of Otolaryngology-Head and Neck Surgery, Stanford University School of Medicine, Stanford, CA 94305, USA

**Keywords:** chronic rhinosinusitis, eosinophilic inflammation, biomarkers, eosinophil cationic protein, eosinophil peroxidase, major basic protein, eosinophil-derived neurotoxin, endotype, nasal polyps, type 2 inflammation

## Abstract

**Background/Objectives**: Chronic rhinosinusitis (CRS) is a heterogeneous inflammatory condition increasingly understood through endotyping, with eosinophilic CRS (eCRS) representing a clinically distinct and often refractory phenotype. Eosinophils exert their pathogenic effects in part through the release of four highly cationic granule proteins—major basic protein (MBP), eosinophil cationic protein (ECP), eosinophil peroxidase (EPX), and eosinophil-derived neurotoxin (EDN)—each with distinct biological activities and measurable tissue and systemic expression profiles. Despite growing interest in precision medicine approaches to CRS management, the clinical utility of these granule proteins as biomarkers has not been systematically characterized. This systematic review evaluates the diagnostic and prognostic utility of eosinophil granule proteins as biomarkers in CRS. **Methods**: This systematic review was conducted per PRISMA (Preferred Reporting Items for Systematic reviews and Meta-Analyses) 2020 guidelines and prospectively registered in PROSPERO (CRD420261399189). PubMed, Embase, Web of Science, and Cochrane Library were searched for studies measuring MBP, ECP, EPX, or EDN in tissue, serum/plasma, nasal secretions, lavage fluid, or nasal swabs in adults with CRS, reporting a predefined clinical outcome (endotype/phenotype discrimination, disease severity, recurrence, or postoperative outcomes). Reported statistics were deemed significant if *p* < 0.05. Meta-analysis was not conducted due to substantial methodological heterogeneity and incomplete outcome reporting across the included studies. **Results**: Of 708 identified references, 71 studies met inclusion criteria. Of the included studies, 73% were cross-sectional studies, and 24% were cohort studies (prospective or retrospective), with 1% classified as case–control and 1% as a randomized controlled trial. ECP was the most extensively evaluated biomarker, evaluated in 65% of the studies meeting inclusion criteria. Several studies evaluated this protein as a tool to discriminate eosinophilic from non-eosinophilic disease and chronic rhinosinusitis with nasal polyps (CRSwNP) from chronic rhinosinusitis without nasal polyps (CRSsNP), with promising results. EPX was less frequently studied but demonstrated a high diagnostic accuracy, including strong performance in minimally invasive nasal secretion and swab sampling. Several studies demonstrated that granule proteins correlated more consistently with objective radiographic or endoscopic severity than with patient-reported symptom scores. ECP showed reproducible prognostic value for postoperative recurrence and disease control. **Conclusions**: Eosinophil granule proteins are promising biomarkers for endotyping and prognostication in CRS. Further work is needed to compare the diagnostic accuracy of these proteins directly, to standardize specimen handling and quantification methods, and to establish threshold values suitable for clinical use. Ultimately, large-scale prospective studies will be required to determine whether these biomarkers can reliably prognosticate clinical outcomes and inform selection of candidates for biologic therapy.

## 1. Introduction

Chronic rhinosinusitis (CRS) is a heterogeneous inflammatory disease of the sinonasal mucosa that significantly impacts patients’ quality of life, impairs healthcare utilization, and increases the economic burden [1]. Persistent sinonasal inflammation causes symptoms including nasal obstruction, rhinorrhea, facial pressure, and hyposmia, which can often lead to the need for repeated medical or surgical interventions. Historically, CRS has been classified phenotypically based on the presence (CRSwNP) or absence (CRSsNP) of nasal polyps. This phenotypic categorization was used as a proxy for the inflammatory endotype, with CRSwNP indicating Th2 cytokine-driven inflammation, whereas CRSsNP represented non-Th2-driven inflammation [2,3]. However, increasing evidence suggests that these phenotypes are inadequate in categorizing the complex underlying inflammatory pathophysiology of the disease, which is better represented by an inflammatory endotype.

Endotype-driven disease classification, specifically the distinction between eosinophilic CRS (eCRS) and non-eosinophilic CRS, is more relevant than ever before in the era of new biologic medications available to treat CRS. Four FDA-approved biologic medications exist to treat CRSwNP, all of which specifically target eCRS. eCRS is characterized predominantly by type 2 inflammation involving eosinophils, immunoglobulin E (IgE), and interleukin (IL)-4, IL-5, IL-13 [4], manifesting clinically as extensive sinonasal inflammation, higher rates of nasal polyp formation, greater symptom burden, and a higher risk for postoperative complications such as recurrence and revision surgery [5,6]. In contrast, non-eosinophilic CRS is more commonly associated with type 1 or type 3 inflammation, with a predominance of neutrophils and cytokines such as interferon-γ (IFN-γ), tumor necrosis factor-α (TNF-α), IL-17, IL-22, and IL-8. Clinically, non-eosinophilic CRS exhibits higher success rates for endoscopic sinus surgery (ESS), lower rates of postoperative recurrence, and a better response to low-dose macrolide antibiotics rather than the currently available biologic therapies targeting type 2 inflammation [5,6,7]. The marked differences in inflammatory pathways, clinical disease manifestation, and therapeutic responsiveness between eCRS and non-eCRS underscore the importance of accurately identifying the endotype of chronic rhinosinusitis to facilitate precise medical intervention and treatment strategies.

Eosinophils exert their pathogenic effects in part through the release of four highly cationic preformed granule proteins: major basic protein (MBP), eosinophil cationic protein (ECP), eosinophil peroxidase (EPX), and eosinophil-derived neurotoxin (EDN). Each possesses distinct biological functions and potential utility as a biomarker of eosinophilic inflammation. MBP is a major constituent of the eosinophil granule crystalloid core and is highly cytotoxic to airway epithelium, contributing to epithelial injury and tissue remodeling following eosinophil degranulation [7,8]. ECP, also known as RNase3, is a ribonuclease with potent cytotoxic and immunomodulatory properties. It promotes epithelial damage, mucus hypersecretion, and activation of inflammatory cells and has been widely investigated as a marker of eosinophil activation [9]. EPX is a heme-containing peroxidase enzyme that is released during active eosinophil degranulation events, thus making it an attractive indicator of eosinophilic disease. EPX, also referred to in the literature as EPO, catalyzes the production of reactive oxygen species, amplifying oxidative tissue injury and local inflammatory responses during eosinophil degranulation [9,10,11]. EDN, less commonly referred to as RNase2, is another eosinophil-derived ribonuclease that is released upon eosinophil degranulation and functions as an alarmin by activating dendritic cells and promoting type 2 immune responses [7,9]. EPX and MBP-2 are the only eosinophil-derived granule proteins (EDGPs) uniquely expressed by eosinophils and not by other cells [7]. Throughout this review, EPX refers exclusively to eosinophil peroxidase; it should be noted that some older literature has used the same abbreviation, or the synonym ‘eosinophil protein X,’ to refer to EDN, an unrelated naming convention not utilized in this review.

Eosinophilic inflammation is a hallmark of type 2 CRS and has traditionally been quantified using tissue eosinophil counts from sinonasal biopsy specimens. Histopathologic tissue eosinophilia, commonly defined as ≥10 eosinophils per high-power field (hpf), is associated with more severe disease and higher rates of post-operative recurrence [12]. Biopsy, however, is invasive and not routinely performed outside of an operative setting, prompting interest in minimally invasive alternatives that reflect the same underlying inflammatory endotype. Eosinophil granule proteins are attractive candidates: they are detectable in sinonasal tissue, nasal secretions, mucus, lavage fluid, and peripheral blood, allowing for sampling without surgical biopsy [8,10,13,14].

While many studies have evaluated the diagnostic and prognostic utility of eosinophil granule proteins in CRS, the most current data have not, to our knowledge, been systematically synthesized. The existing literature is heterogeneous with respect to the proteins evaluated, specimen types, sampling methods, disease definitions, and clinical outcomes assessed, which limits assessment of overall clinical applicability. The relationships between eosinophil granule protein levels and established markers of type 2 inflammation, disease severity, and postoperative recurrence also remain incompletely characterized. This systematic review therefore evaluates the current evidence for the four major eosinophil granule proteins as biomarkers in CRS, with specific attention to their associations with eCRS, disease severity, and postoperative recurrence, and to the feasibility of minimally invasive sampling as an alternative to tissue eosinophil quantification.

## 2. Materials and Methods

### 2.1. Study Design

This systematic review was conducted in accordance with the Preferred Reporting Items for Systematic Reviews and Meta-Analyses (PRISMA) 2020 guidelines [15]. The review protocol was prospectively developed prior to study selection and data extraction and submitted for registration in the International Prospective Register of Systematic Reviews (PROSPERO), and registration was obtained (Registration No. CRD420261399189). The objective of this review was to evaluate the diagnostic and prognostic utility of eosinophil granule proteins, including major basic protein (MBP), eosinophil cationic protein (ECP), eosinophil peroxidase (EPX), and eosinophil-derived neurotoxin (EDN) as biomarkers of disease characterization, severity assessment, and postoperative outcomes.

### 2.2. Eligibility Criteria

A comprehensive literature search was performed in PubMed, Embase, Web of Science, and the Cochrane Library. The search aimed to identify studies evaluating the diagnostic and prognostic utility of eosinophil granule proteins in patients with CRS. The search terms included combinations of keywords and subject headings [(sinusitis OR nasal polyps OR chronic rhinosinusitis OR chronic sinusitis OR rhinosinusitis OR nasal polyp OR nasal polyposis) AND (eosinophil granule proteins OR eosinophil granule protein OR eosinophil cationic protein OR RNase 3 OR eosinophil major basic protein OR major basic protein OR eosinophil-derived neurotoxin OR eosinophil derived neurotoxin OR RNase 2 OR eosinophil peroxidase)]. The search included articles published from 1 January 2000, through 12 June 2026. In addition, the reference lists of all included studies, relevant review articles, and other pertinent publications were manually screened to identify further eligible studies. The most recent literature search was conducted on 12 June 2026. The complete database-specific search strategies, including controlled vocabulary, keywords, field tags, truncation operators, filters, and search dates, are provided in Supplementary Material S1: Complete database-specific search strategies.

### 2.3. Study Selection

**Figure 1:**
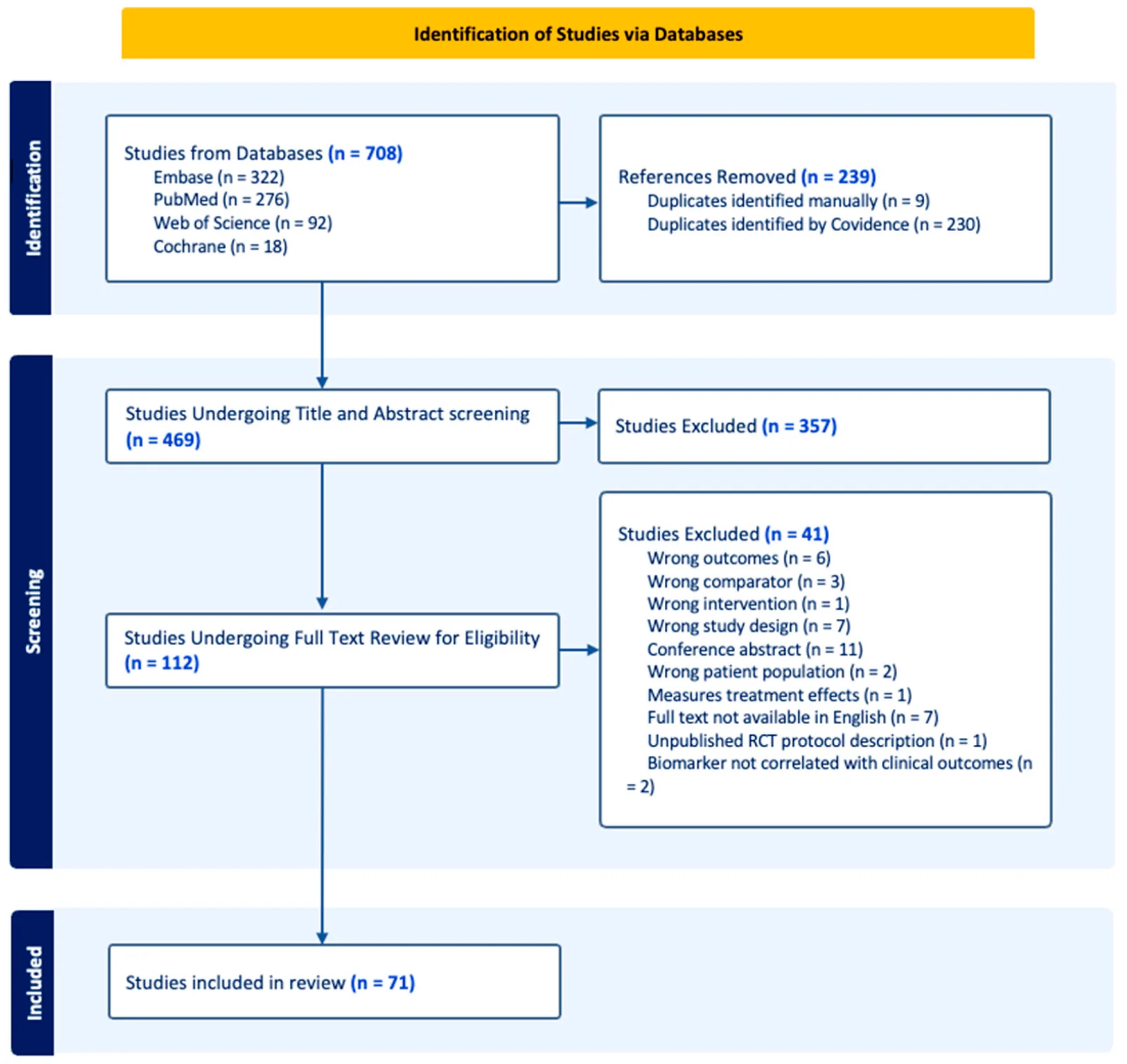
Flowchart of the literature search and study selection process. This PRISMA flow diagram illustrates the identification, screening, eligibility assessment, and inclusion of studies evaluating eosinophil granule proteins as biomarkers in chronic rhinosinusitis.

Three reviewers independently screened the titles, abstracts, and full texts of all retrieved studies for eligibility through Covidence 2026 (Veritas Health Innovation, Melbourne, Australia) (SNF, JBS, JKC). Studies meeting the inclusion criteria or lacking sufficient information for exclusion underwent full-text review. Full-text articles were independently assessed by two reviewers to determine final eligibility. Any conflicts arising during the title and abstract screening or full-text review stages were resolved through discussion involving all three authors until consensus was reached. The study selection process was documented using a Preferred Reporting Items for Systematic Reviews and Meta-Analyses (PRISMA) flow diagram [Figure 1]. The methodological quality of included studies was independently assessed by two independent reviewers using the Newcastle-Ottawa Scale (NOS).

### 2.4. Data Extraction

The following variables were collected: study title, authors, publication year, study design, study population, sample size, patient demographics (age and sex), chronic rhinosinusitis phenotype (CRSwNP or CRSsNP), eosinophilic CRS status (when reported), diagnostic criteria for CRS, specimen source (tissue, serum, plasma, nasal secretions, nasal lavage fluid, or nasal swab), relevant comorbidities (asthma, aspirin sensitivity, aspirin-exacerbated respiratory disease [AERD], and allergic rhinitis/atopy), eosinophil granule protein evaluated (major basic protein [MBP], eosinophil cationic protein [ECP], eosinophil-derived neurotoxin [EDN], or eosinophil peroxidase [EPX]), biomarker quantification method, comparator groups, disease severity measures, clinical outcomes assessed, and principal study findings. For each included study, quantitative data on eosinophil granule protein expression and its associations with CRS phenotype, disease severity, postoperative outcomes, disease recurrence, and relevant comorbidities were extracted when available. Reported statistics were deemed significant if *p* < 0.05. eCRS is defined by the histologic threshold of ≥10 eosinophils per high-power field (eos/hpf), consistent with prior literature [12]. Where an included study used this criterion, no additional note is made. However, where a study classified eCRS using an alternative method, the specific criterion used is stated. eCRS, type 2 CRS, CRSwNP, and eosinophilic CRSwNP are related but non-synonymous classifications; each is reported here using the terminology and definition applied in the original study and is not treated as interchangeable with the others. It is also important to note that to account for a broader time range, we included studies that did not specify endotype of CRS—in these scenarios, this histologic definition was also not utilized. Heterogeneity in study populations, biomarker assays, specimen sources, and outcome measures necessitated a narrative synthesis of key findings rather than a quantitative meta-analysis.

## 3. Results

### 3.1. Search Results

The literature search yielded 708 references: 322 from Embase, 276 from PubMed, 92 from Web of Science, and 18 from Cochrane Library. A total of 239 duplicates were identified and removed, of which 9 were identified manually and 230 were identified using Covidence systematic review software (Veritas Health Innovation, Melbourne, Australia), leaving 469 unique studies for title and abstract screening. From these, 357 were excluded, and 112 studies underwent full-text review for eligibility. Forty-one studies were excluded at the full-text stage for the following reasons: conference abstract (*n* = 11), full text not available in English (*n* = 7), wrong study design (*n* = 7), wrong outcomes (*n* = 6), wrong comparator (*n* = 3), biomarker not correlated with clinical outcomes (*n* = 2), wrong patient population (*n* = 2), measures treatment effects (*n* = 1), unpublished randomized control trial (RCT) protocol description (*n* = 1), and wrong intervention (*n* = 1). No studies were ongoing or awaiting classification at the close of screening. A total of 71 studies met inclusion criteria and were included in the qualitative synthesis. The workflow of the study selection process is outlined in Figure 1.

### 3.2. Characteristics of Included Studies

Seventy-one studies met the inclusion criteria [14,15,16,17,18,19,20,21,22,23,24,25,26,27,28,29,30,31,32,33,34,35,36,37,38,39,40,41,42,43,44,45,46,47,48,49,50,51,52,53,54,55,56,57,58,59,60,61,62,63,64,65,66,67,68,69,70,71,72,73,74,75,76,77,78,79,80,81,82,83,84] (Table 1), published between 2000 and 2026, with more than a third (*n* = 25, 35%) published in the last three years (2023–2026), reflecting growing interest in eosinophil granule proteins as CRS biomarkers. In terms of study design, 52 studies (73%) used a cross-sectional comparative design, 17 (24%) were cohort studies (prospective or retrospective), 1 (1%) was a case–control study, and 1 (1%) was a randomized controlled trial. ECP was the most frequently evaluated biomarker, assessed in 46 studies (65%), followed by MBP (13 studies, 18%), EPX (10 studies, 14%), and EDN (8 studies, 11%); several studies evaluated more than one granule protein (Table 2). Regarding specimen source, tissue was the most commonly used, evaluated in 48 studies (68%), followed by nasal secretions/mucus (30 studies, 42%), and serum, plasma, or peripheral blood (29 studies, 41%) (Table 3); many studies assessed more than one specimen type. Among the nasal secretion/mucus collections, a wide variety of techniques were used to obtain the specimen sample, including cytology brushing, an absorption technique using cotton wool sticks or sponges, a sterile sinus secretion collector, nasal secretion smear, and nasal swabs or cytology brushes.

### 3.3. Quality Assessment

The Newcastle-Ottawa Scale (NOS) was used to assess the methodological quality and risk of bias of the included case–control and cohort studies [86]. For cross-sectional studies, the adapted NOS-xs scale for cross-sectional studies was used [87]. Two reviewers independently assessed each study using the appropriate assessment tool, and any disagreements were resolved through discussion and consensus. Studies with total scores of 7–9 were classified as having a low risk of bias, scores of 4–6 as having a moderate risk of bias, and scores of 0–3 as having a high risk of bias. Overall, 17 (23.9%) studies were rated as having low risk of bias, 53 (74.6%) studies as having moderate risk of bias, and 1 (1.4%) study as having high risk of bias based on the total score. Table 1 summarizes the risk-of-bias assessment for the included studies.

### 3.4. Diagnostic Utility of Eosinophil Granule Proteins

Numerous studies evaluated the utility of eosinophil granule proteins for distinguishing patients with type 2 inflammation. In older studies, this involved phenotypic classification (CRSwNP versus CRSsNP), though many more recent studies evaluated the role of using these proteins to distinguish eCRS versus non-eCRS. Generally, this endotypic diagnosis was made using the current gold standard of histopathology demonstrating a specific cut-off of eosinophils per high-power field. Key diagnostic-performance findings are summarized in Table 4.

#### 3.4.1. Eosinophil Cationic Protein

ECP was the most extensively studied biomarker for endotype discrimination. Across studies directly comparing eCRS with non-eCRS, ECP measured in tissue and secretions was significantly associated with eCRS status in multiple independent cohorts. At the transcript level, ECP mRNA was selectively upregulated in eosinophilic CRSwNP compared with non-eosinophilic CRSwNP (*p* = 0.001), CRSsNP (*p* = 0.001), and controls (*p* = 0.001), with a corresponding increase in protein expression. ECP expression was similarly elevated in eosinophilic CRSwNP within a broader Chinese CRS cohort that also included CRSsNP and non-eosinophilic CRSwNP (*p* = 0.007) [64], and in a cohort comparing eCRSwNP to non-eCRSwNP and controls [35]. ECP differed significantly between eosinophilic from non-eosinophilic disease across fungal rhinosinusitis and eosinophilic CRSwNP subgroups, using a substantially higher threshold of ≥120 eosinophils/hpf [48], and across type 2 versus non-type 2 CRS defined by a peripheral-blood/allergy composite (EPOS/EUFOREA criteria) rather than tissue histology [70]; a third study reported this association without specifying its eosinophil classification criteria [78].

Specificity for the eosinophilic endotype was also supported in a study in which ECP in nasal secretions was significantly elevated in eosinophilic disease among non-neutrophilic CRS endotypes, while myeloperoxidase (MPO) was more strongly associated with neutrophilic disease [38]. Serum ECP differentiated type 2 from non-type 2 CRS (*p* = 0.039), with further elevation in the uncontrolled type 2 disease (OR: 16.7, 95% CI: 1.7–161.6, *p* = 0.015) (classified patients by a peripheral-blood/allergy composite rather than tissue eosinophilia) [70]. Nasal-secretion ECP performed well as a minimally invasive diagnostic tool, distinguishing eCRS from non-eCRS with an area under the curve (AUC) of 0.823 (cutoff 73.88 μg/mL; sensitivity 85.4%, specificity 70.3%, *p* < 0.0001) [37]. Though other studies reported ROC curves for diagnostic accuracy [38,44,46,70,84], this was the only study to report a formal measure of diagnostic accuracy with a defined threshold; the remaining studies reported between-group differences without accompanying discriminative statistics. Reported concentrations also varied by specimen type and assay, with tissue values expressed in mg/g and secretion values in μg/mL, such that thresholds are not directly transferable across sampling methods.

When comparing CRSwNP to CRSsNP, tissue ECP was significantly higher in the CRSwNP phenotype across multiple independent cohorts [31,59,61,73]. Tissue ECP concentrations were also elevated in untreated nasal polyp tissue relative to control mucosa (*p* = 0.003 [17]; *p* < 0.001 [18]), and serum ECP mirrored this pattern (*p* = 0.014 [84]). Two studies described an eosinophil severity gradient, with the highest ECP concentrations observed in AERD-associated CRSwNP, followed by CRSwNP, and lowest in CRSsNP [14,51].

Two studies reported type 2 inflammation within CRSsNP patients, indicating that the polyp phenotype does not fully align with the eosinophilic endotype. Significantly higher levels of ECP were reported in patients with Type 2 CRSsNP compared with non-Type 2 CRSsNP (7.86 ± 6.67 mg/g vs. 1.14 ± 1.82 mg/g, *p* < 0.0001) [24]. Similarly, ECP was found to be the only type 2 biomarker significantly elevated in CRSsNP compared with healthy controls [32]. One study found no significant difference in ECP between central-compartment atopic disease (CCAD) and other CRS phenotypes; in that analysis, comparator patients were grouped as a single CRS category without separation into CRSwNP and CRSsNP [45]. Serum ECP was not correlated with tissue eosinophils despite independently predicting postoperative recurrence in the same cohort (OR: 54.8, 95% CI: 2.3–1327.3, *p* = 0.014) [46], indicating that a granule protein’s prognostic value does not guarantee it tracks the histologic reference standard used to define eCRS.

#### 3.4.2. Eosinophil-Derived Neurotoxin

Evidence for EDN was more limited than for ECP but directionally consistent, with the notable feature that EDN outperformed peripheral blood eosinophil counts in two independent cohorts. Serum EDN was significantly associated with eosinophilic CRS status (classified according to the Japanese Epidemiological Survey of Refractory Eosinophilic Chronic Rhinosinusitis JESREC criteria) compared with non-eosinophilic CRS, allergic rhinitis, and other sinonasal disease, with serum EDN levels significantly elevated in eCRS compared to healthy controls (*p* < 0.01); moreover, serum EDN positively correlated with nasal polyp score (NPS) (r = 0.35, *p* < 0.05), JESREC score (r = 0.64, *p* < 0.01), disease stage (r = 0.55, *p* < 0.05), blood eosinophil count (r = 0.78, *p* < 0.01), and blood eosinophil percentage (r = 0.68, *p* < 0.01) [71]. EDN in nasal mucus differentiated symptomatically controlled from uncontrolled CRS, with an odds ratio for uncontrolled CRS of 1.323 (95% CI 1.175–1.659, *p* = 0.004) and an AUC of 0.897, significantly exceeding that of peripheral blood eosinophil counts (AUC 0.869, *p* = 0.042 for the difference) [34]. In addition, mucus EDN predicted olfactory dysfunction (OR 2.162, *p* = 0.027) with an AUC of 0.873 versus 0.764 for blood eosinophils [77]; note that these two studies share overlapping authorship and may derive from overlapping patient recruitment rather than representing independent cohorts [34,77]. A subsequent cross-sectional study corroborated the disease-control finding, with nasal mucus EDN being significantly higher in uncontrolled than controlled CRS (AUC = 0.798, 95% CI: 0.692–0.904, *p* < 0.001; type 2 CRS defined by peripheral blood eosinophil count > 0.24 × 10^9^/L per EPOS2020) [85]. At the tissue level, purified eosinophils isolated from the nasal polyps of eCRS patients (classified via JESREC score ≥11 plus ≥70 eosinophils/hpf) expressed significantly higher EDN than peripheral blood eosinophils from the same patients, indicating local eosinophil activation and degranulation [82]. Two studies proposed candidate diagnostic cutoffs for mucus EDN based on ROC analysis within their own cohorts (398.8 ng/mL [34]; 407.355 ng/mL [77]), but neither was validated in an independent cohort, and no included study compared EDN directly against tissue eosinophil count as a reference standard.

#### 3.4.3. Eosinophil Peroxidase

EPX demonstrated the highest reported AUCs for diagnostic accuracy among the proteins evaluated. Tissue EPX discriminated eosinophilic from non-eosinophilic CRS independent of nasal polyp status, with an AUC of 0.938 (95% CI: 0.835–1.00; cutoff 470 ng/mL; specificity 100%, sensitivity 81.3%, *p* = 0.002) [36]. Minimally invasive nasal mucus sampling produced comparable discrimination: EPX measured by nasal cytology brushing distinguished eCRS from non-eCRS with an AUC of 0.89 (sensitivity 71.4%, specificity 92.9%, *p* = 0.001) [22]. A nasal-swab EPX activity assay and ethmoid tissue biopsy likewise showed significantly elevated EPX in eCRS, as determined by histologic tissue evaluation showing >10 eosinophils per high-powered field (*p* < 0.0001 and *p* < 0.05, respectively) [66]. For phenotypic evaluation, tissue EPX was elevated in CRSwNP as compared to control subjects without CRS (*p* = 0.007) but did not significantly differ between eosinophilic CRSwNP and eosinophilic CRSsNP, suggesting EPX tracks an eosinophilic endotype more consistently than polyp phenotype [36]. No included study reported external validation of an EPX threshold or assessed inter-assay reproducibility.

#### 3.4.4. Major Basic Protein

MBP findings were directionally consistent with the other granule proteins but were reported with less diagnostic-accuracy quantification, and more often as semi-quantitative staining or deposition scores than as continuous concentrations. Comparison of absolute values was limited due to lack of standardization of scoring systems across studies. MBP was significantly elevated in CRS patients compared with non-CRS controls (*p* = 0.0018) [16]. Within CRS groups, MBP was also higher in eosinophilic than non-eosinophilic cohorts in two independent cohorts (*p* <0.05 to *p* < 0.001) using either a >10% eos/hpf threshold or a JESREC-based composite score (≥11 plus ≥70 eosinophils/hpf), respectively [39,68]. For a phenotypic CRSwNP-versus-CRSsNP comparison, MBP-positive cells progressively increased across controls, CRSsNP, CRSwNP uncinate tissue, and CRSwNP nasal polyp tissue (*p* < 0.05 for each stepwise comparison), and eosinophilic CRSwNP showed significantly higher MBP expression than non-eosinophilic CRSwNP (*p* < 0.05 to *p* < 0.001) [39]. This pattern was corroborated by another study reporting that a Th2-predominant subject cluster composed of 100% CRSwNP patients was more strongly associated with a Th2 cytokine signature than the other two clusters (*p* < 0.001) and had significantly higher tissue MBP than both a CRSwNP-free cluster (57% controls/43% CRSsNP; *p* = 0.001) and a CRSsNP-predominant cluster (62.5% CRSsNP; *p* = 0.01) [27]. In patients with CRSwNP, tissue MBP expression was significantly elevated in both nasal polyp and adjacent inferior turbinate tissue compared with normal sinonasal mucosa (*p* < 0.001) but did not differ significantly between diseased tissue samples from the same patients (*p* < 0.102), suggesting eosinophilic inflammation in CRSwNP extends diffusely rather than being localized to polyps [76]. In one cohort, MBP showed no correlation with tissue eosinophils (r = 0.091, *p* = 0.609), despite correlating significantly with both the JESREC eosinophilic score and blood eosinophil counts in the same study [68], demonstrating that MBP’s relationship with other type 2 markers does not necessarily extend to the histologic gold standard. Overall, MBP evidence in this review remained at the association stage: no study reported an AUC, a proposed threshold, or external validation.

### 3.5. Comparison of Biological Specimen Sources

Eosinophil granule proteins were measured across a range of specimen types including sinonasal tissue, nasal secretions/mucus, and serum. Studies demonstrated meaningfully different diagnostic performance depending on the compartment sampled.

#### 3.5.1. Nasal Tissue

Tissue was the specimen type most frequently sampled across all four biomarkers and produced the most consistent discriminatory signal in the dataset, including the highest reported AUC overall (tissue EPX, AUC 0.938, 95% CI 0.835–1.00) [36]. ECP, EPX, MBP, and EDN were each reproducibly and often significantly elevated in eosinophilic CRS/CRSwNP tissue compared with non-eosinophilic disease and healthy controls [16,17,18,23,34,38,42,67,72,75,81], and tissue eosinophil counts (typically ≥10 eosinophils/hpf) served as the reference standard used to define eosinophilic-CRS status against which other biomarkers and specimen types were validated [36,41,47,66]. Beyond diagnostic discrimination, tissue biomarker levels correlated with radiographic and symptomatic disease severity [41,74] and predicted postoperative outcomes, including polyp recurrence and residual inflammation after ESS [20,21,44,75]. Findings were not uniformly positive, however: tissue ECP failed to reliably diagnose CRS in some cohorts [83] and showed no discrimination in another study comparing central-compartment disease to other CRS endotypes [45]. Similarly, tissue EPX did not differ between CCAD and eCRS despite differences in eosinophil density in a separate cohort (*p* = 0.183) [65].

#### 3.5.2. Nasal Secretions

Measurement of protein levels within nasal secretions is a promising minimally invasive alternative to tissue sampling. Within the nasal secretions category, studies collected specimens via various mechanisms including nasal swabs, cytology brushes (with purposeful separation of nasal secretions and epithelial cells), nasal lavage, and absorbent sponges. The area of collection of secretions within the nasal cavity also differed, though a majority of these studies opted for collection of secretions within the middle meatus, with the exception of lavage fluid, given that this non-specifically collects nasal secretions. Nasal-secretion ECP, EPX, and EPX activity all discriminated eCRS from non-eCRS with high accuracy (AUC 0.823–0.89, sensitivity 71–85%, specificity 70–93%) [22,37,38,66], and these specimens also correlated with tissue-based eosinophilic inflammation [38]. Nasal mucus MBP was significantly different between CRS and controls in three reports, detected in 87–92% of CRS patients versus 0–5% of controls (sensitivity 87–92%, specificity 100%, *p* < 0.001 in all three) [55,56,63]; two of these come from the same research group and may reflect overlapping or sequentially expanded patient populations.

Lavage ECP was significantly elevated in nasal polyposis versus control patients (38.1 vs. 16.1 ng/mL, *p* < 0.001) [25], higher in CRSwNP than in controls and CRSsNP (*p* < 0.001 and *p* < 0.01, respectively) [14], elevated in nasal polyposis compared to controls (60.6 vs. 2.4 ng/mL, *p* < 0.001) [69], and higher in asthmatic than non-asthmatic CRSsNP (36.1 vs. 2.7 μg/L, *p* = 0.0001) [72]. One study reported EPX lavage expression significantly higher in CRSwNP and CRSwNP + asthma groups (*p* < 0.01 and *p* < 0.001, respectively) than controls and correlated EPX expression between nasal lavage and induced sputum, supporting lavage as a reasonable alternative means of collecting nasal secretions [81].

#### 3.5.3. Serum ECP

Several studies did find significant differences in serum ECP: higher in CRS without allergic rhinitis than controls (21.9 vs. 9.7 μg/L, *p* = 0.0002) [40]; an independent predictor of postoperative recurrence in two studies (OR: 1.283, 95% CI: 1.044–1.576, *p* = 0.018) [44] and OR: 54.8, 95% CI: 2.3–1327.3, *p* = 0.014 [46]); significantly higher in refractory than primary eosinophilic nasal polyps (*p* = 0.001) [60]; an independent predictor of uncontrolled Type 2 disease (OR: 16.7, 95% CI: 1.7–161.6, *p* = 0.015) [70]; significantly higher in CRSwNP patients with comorbid asthma (68.8 vs. 36.3 μg/L, *p* = 0.015) and an independent predictor of asthma comorbidity (OR: 1.01, *p* = 0.037) [79]; significantly higher in patients with postoperative residual inflammation (62.7 vs. 38.4 μg/L, *p* = 0.039) [80]; and significantly higher in CRSwNP than CRSsNP (*p* = 0.014) with an independent recurrence association (OR: 2.77, *p* = 0.003) [84].

However, a comparable number of studies measuring ECP in serum or unspecified peripheral blood found associations that did not reach statistical significance, even in cohorts where tissue or nasal-secretion ECP was clearly elevated. Nasal lavage ECP was significantly elevated in CRSwNP (38.1 vs. 16.1 ng/mL, *p* < 0.001), while serum ECP in the same patients was unchanged [25]. Similarly, despite a near-significant tissue difference in ECP between CRSwNP and control groups (*p* = 0.0506), serum ECP did not significantly differ between CRSwNP and controls (4.1 vs. 5.4 μg/L) [83]. Serum ECP showed no significant association with atopy, symptom severity, or recurrence in another cohort [42]. Circulating ECP was six-fold higher in CRSwNP than controls but did not reach significance [29]. Nasal ECP rose significantly after surgery relative to controls, while serum ECP changes were not statistically significant in the same cohort [54]. A cohort with clearly elevated nasal ECP (208.2 vs. 57.2 ng/mL, *p* < 0.001) explicitly reported no significant serum ECP correlation [67].

Additionally, mucus EDN also directly outperformed blood eosinophil counts for predicting disease control status (AUC 0.897 vs. 0.869, *p* = 0.042) [34] and olfactory dysfunction (AUC 0.873 vs. 0.764, *p* = 0.024) [77].

### 3.6. Association with Disease Severity

#### 3.6.1. SNOT-22/SNOT-20

Several studies found significant positive correlations between granule protein levels and patient-reported symptom burden: tissue MBP correlated with SNOT-22 (r = 0.48, *p* = 0.005) [74]; tissue EPX correlated with SNOT-22 (*p* = 0.041) [36]; serum ECP correlated with SNOT-20 (r = 0.875, *p* = 0.001) [40] and SNOT-22 (*p* < 0.05) [84]. Serum ECP was significantly higher in patients with uncontrolled versus controlled disease, alongside significantly worse SNOT-22 scores in the same cohort [84]. Mucus EDN was significantly higher in uncontrolled CRS and correlated with significantly worse SNOT-22 in two studies (r = 0.325, *p* < 0.01) [85], (*p* < 0.001) [34]. Higher baseline mucus EPX predicted greater SNOT-22 improvement after treatment with dupilumab (ρ = −0.70, *p* = 0.038) [23]. However, several studies found no significant direct correlation between the biomarker itself and SNOT-22: nasal EPX [22], swab EPX [66], and serum ECP [70] (despite T2 CRS patients having higher baseline SNOT-22 than non-T2 patients, 51 vs. 39.5, *p* = 0.044). Additionally, Harmon et al. found that while ESS significantly improved SNOT-22 (*p* < 0.0001), the eosinophilic biomarker itself (ECP) did not significantly change (*p* = 0.15 [32].

#### 3.6.2. Lund-MacKay CT Score

ECP correlated significantly with computed tomography (CT) scores across multiple independent cohorts, whether measured in serum, nasal secretions, or tissue (r = 0.42–0.853, *p* ≤ 0.01) [40,67,75], and higher preoperative ECP was similarly linked to higher CT scores in two recurrence-prediction cohorts [21,80]. However, a few other ECP studies found no significant CT correlation in serum ECP [19,42,46] and mucus ECP [52,69]. Tissue MBP correlated significantly with CT scores in two studies (r = 0.516–0.54, *p* < 0.05) [39,74], but a third study failed to show a significant correlation (r = 0.117, *p* = 0.509) [68]. Tissue EPX and mucus EDN correlated significantly with CT score in the cohorts that assessed them [34,36]. A Th2-predominant cluster showed significantly higher CT scores than other inflammatory clusters (*p* ≤ 0.05) [27].

#### 3.6.3. Lund-Kennedy Endoscopy Score

ECP correlated with Lund-Kennedy Endoscopy scores across secretion, serum, and multigroup comparisons [37,38,80,84,85], while EPX showed one significant correlation (rs = 0.440, *p* < 0.05 [66]). MBP showed no significant association with postoperative Lund-Kennedy scores [39].

#### 3.6.4. Nasal Polyp Score

Significant positive associations were found for serum EDN (correlated with nasal polyp score) [71], serum ECP (higher polyp score in the residual-inflammation group, 5.9 vs. 5.2, *p* = 0.033) [80], and eosinophil CD69 activation marker (r = 0.624, *p* < 0.05) [82]. Notably, Bachert et al. explicitly reported that ECP was not significantly correlated with polyp score despite being strongly associated with asthma comorbidity in the same cohort [19].

#### 3.6.5. Symptom Scores

Nasal lavage ECP correlated significantly with several individual symptom domains—nasal obstruction (*p* = 0.003), congestion (*p* = 0.009), rhinorrhea (*p* = 0.009), and overall Visual Analogue Scale (VAS) score (*p* = 0.0390) [69], and multi-group VAS comparisons across neutrophilic/eosinophilic CRS subgroups were also significant (*p* = 0.0041) [38]. Similarly, in a large postsurgical CRS cohort, nasal mucus EDN was significantly associated with bothersome congestion (OR 4.09, *p* = 0.031) and rhinorrhea (OR 3.61, *p* = 0.011), while MBP was associated with sneezing (OR 2.71, *p* = 0.034) [57]. Total nasal symptom score (TNSS) correlated moderately with mucus ECP [26], and nasal polyps overall showed higher symptom scores than CRS without polyps, particularly for congestion and loss of smell [73]. In contrast, atopy status showed no significant VAS association despite being present in nearly half the CRSwNP cohort studied [42], and mucus ECP was not directly correlated with total nasal symptom score (TNSS) in a separate AERD-focused cohort, even though AERD patients (who had the highest ECP) also showed the greatest overall inflammatory mediator expression [52].

### 3.7. Prognostic Utility

Findings across recurrence, revision surgery, persistent inflammation, and disease control are summarized in Table 5. The broader biomarker-specific evidence across diagnostic, disease severity, disease control, and prognostic outcomes is summarized in Table 6.

#### 3.7.1. Disease Recurrence

Across multiple cohorts, elevated tissue and serum ECP consistently correlated with and independently predicted postoperative polyp recurrence after ESS [20,21,40,44,46,67,84]. In a prospective CRSwNP cohort, tissue ECP independently predicted postoperative recurrence, and a composite model incorporating ECP with IL-5, anti-dsDNA IgG, asthma status, and radiographic severity significantly improved predictive performance over clinical variables alone (AUC = 0.89 combined model vs. 0.78 biomarker model) [20]. A subsequent report from the same investigative group extended this cohort, incorporating the original 94 patients as a training set alongside 61 additional patients as a validation set; tissue ECP increased stepwise across low-, intermediate-, and high-risk recurrence groups (248, 826, and 2290 ng/mg, *p* < 0.0001), with high-risk patients demonstrating a hazard ratio of 9.8 for recurrence relative to low-risk patients, and the composite risk score maintaining strong discrimination in a validation cohort (AUC = 0.89 training, 0.76 validation) [21]. Serum and nasal ECP showed comparable prognostic strength in independent cohorts, with recurrence groups demonstrating significantly higher ECP than non-recurrence groups (32.8 vs. 19.8 µg/L, *p* = 0.036 [40]; 287.7 vs. 131.9 ng/mL, *p* < 0.001 [67]), and multivariable models identifying ECP as an independent predictor of recurrence following ESS (OR: 1.283, 95% CI: 1.044–1.576, *p* = 0.018, AUC = 0.680 [44]; OR: 54.8, 95% CI: 2.3–1327.3, *p* = 0.014, AUC = 0.729, NPV 94.7% [46]; OR: 2.77, *p* = 0.003, AUC = 0.77 alone rising to 0.84 combined with total IgE [84]).

Consistent with these findings, recurrence occurred significantly more often in patients with a biologically active type 2 CRSsNP endotype than in non–type 2 disease (19.0% vs. 9.2%, *p* < 0.05) [24]. Notably, one study directly compared biopsy site and found that ECP measured in uncinate tissue was a substantially stronger predictor of recurrence than ECP from nasal polyp tissue itself (uncinate ECP ≥ 290 ng/mg: HR 2.84, 95% CI: 1.14–7.03, *p* = 0.025; nasal polyp ECP: ≥290 ng/mg: HR 1.74, 95% CI 0.74–4.13, *p* = 0.207) [75]. Additionally, Nordström et al. found tissue EDN levels to be significantly elevated in patients with recurrent polyps within a lipid-mediator-driven inflammatory signature (elevated LTE4, PGD2, 15(S)-HETE, and IL-13), and previous NP surgery and AERD were also significantly more common amongst these patients [49].

However, not all studies supported eosinophil granule proteins as recurrence biomarkers. Serum ECP showed no association with recurrence risk or disease severity in atopic versus non-atopic CRSwNP [42], and tissue ECP did not significantly differ between CRSwNP patients and controls in a Chinese cohort, nor did it correlate with clinical outcomes [83]. Similarly, tissue MBP was not associated with postoperative recurrence despite correlating significantly with JESREC score and blood eosinophils in one study [68].

#### 3.7.2. Revision ESS

Elevated tissue ECP in patients with a biologically active type 2 CRSsNP endotype was associated with a significantly higher recurrence rate than non–type 2 disease (19.0% vs. 9.2%, *p* < 0.05), and among patients who recurred, 80.6% ultimately required revision surgery [24]. In contrast, while higher intraoperative EPX activity correlated with increased postoperative antibiotic use (r = 0.37, *p* = 0.01), oral corticosteroid use (r = 0.44, *p* = 0.002), and biologic initiation (r = 0.34, *p* = 0.018), it was not significantly associated with the need for revision surgery (r = 0.17, *p* = 0.24) [47]. Similarly, in a cross-sectional study in which refractory CRSwNP was defined as patients requiring revision ESS after poor response to medical therapy, tissue ECP levels were elevated in both primary and refractory disease (defined as disease requiring revision FESS after failure of maximal postoperative medical therapy) compared with controls, but did not differ significantly, indicating that ECP alone was not a reliable marker of revision disease [60]. In addition, while AERD patients had significantly more prior revision surgeries and markedly higher tissue ECP than CRSwNP patients (~5-fold, *p* < 0.001), ECP itself was not modeled as a predictor of surgical history [14].

#### 3.7.3. Persistent Inflammation

Eosinophilic inflammation appeared to persist despite surgical intervention and comorbid disease control in two cohorts. In patients with CRSwNP undergoing ESS, patient-reported symptoms and radiographic disease severity improved postoperatively, and IL-13 and periostin levels decreased significantly (67% and 66% reductions, respectively; both *p* < 0.01); however, ECP levels remained unchanged (*p* = 0.15) [32]. Similarly, in eosinophilic CRSwNP patients evaluated for postoperative residual inflammation at 3 months, serum ECP was significantly higher among those with residual inflammation than good responders (62.7 vs. 38.4 µg/L, *p* = 0.039), though on multivariate analysis ECP did not independently predict residual inflammation, while comorbid asthma did [80].

#### 3.7.4. Disease Control Status

Eosinophil-derived biomarkers were also associated with clinical disease control. Nasal mucus EDN was significantly higher in patients with uncontrolled versus controlled CRS (425.36 vs. 214.73 pg/mL, *p* < 0.001) and, as a standalone biomarker, demonstrated good discriminatory ability for identifying uncontrolled disease (AUC = 0.798, 95% CI: 0.692–0.904, *p* < 0.001) [85]. Furthermore, mucus EDN was an independent predictor of uncontrolled CRS (OR: 1.323, 95% CI: 1.175–1.659, *p* = 0.004) [34]. In a small prospective pilot cohort, nasal MBP and EDN both rose significantly during acute CRS exacerbations relative to each patient’s own baseline, alongside worsening SNOT-22 scores, supporting these biomarkers as trackers of within-patient disease-control state over time [58]. Additionally, nasal MBP levels rose significantly during acute symptomatic exacerbations of CRSwNP, compared with baseline (*p* = 0.02) [28]. Serum ECP similarly predicted postoperative uncontrolled disease, but only within the Type 2 endotype: patients with T2 CRS and serum ECP ≥ 17 µg/L had a markedly increased risk of uncontrolled disease after surgery (OR: 16.7, 95% CI: 1.7–161.6, *p* = 0.015; HR: 11.3, 95% CI: 1.5–87.3, *p* = 0.020; AUC = 0.715, NPV 96.8%), whereas serum ECP had no predictive value in non-T2 CRS [70]. Tissue ECP expression followed a similar pattern by phenotype, with eosinophilic CRSwNP showing the highest ECP staining intensity and significantly poorer 1-year postoperative disease control: uncontrolled disease was observed in 93% of eosinophilic CRSwNP patients, compared with 27% of non-eosinophilic CRSwNP patients and 0% of CRSsNP patients [78].

## 4. Discussion

### 4.1. Eosinophil Granule Proteins as Endotype and Phenotype Discriminators

This systematic review synthesized evidence from 71 studies evaluating the four major eosinophil granule proteins (MBP, ECP, EPX, and EDN) as biomarkers of eosinophilic inflammation in CRS. Importantly, evidence in this review reflects distinct stages of biomarker development, not equivalent proof of clinical utility. We distinguish (1) association, a significant difference in biomarker levels between groups; (2) correlation, an association with a continuous severity or inflammation measure; (3) diagnostic/prognostic discrimination, ROC/AUC; (4) threshold determination, a proposed cutoff for classification or outcome prediction; (5) external validation, performance confirmed in an independent cohort; and (6) clinical utility, biomarker-guided decisions shown to improve outcomes. Most included studies reached only the association or correlation stage; few reported discrimination or proposed thresholds; validation in an independent cohort was rare; and no study demonstrated clinical utility.

ECP was the most extensively studied biomarker and showed the most consistent evidence for associating both eosinophilic endotype and nasal-polyp phenotype across specimen sources. Despite being evaluated in fewer studies, EPX produced the strongest quantitative evidence for discrimination, with an AUC of 0.938 for tissue EPX [36] and an AUC of 0.89 [22] for nasal secretion EPX. EDN also showed discriminatory potential, including outperforming peripheral blood eosinophil counts in predicting disease control status and olfactory dysfunction, though these findings come from reports with overlapping authorship [34,77]. MBP findings were supportive of the same overall pattern as a marker of both the eosinophilic endotype and polyp phenotype; however, limited reporting of diagnostic performance metrics limits its current clinical applicability. Overall, ECP and EPX have the strongest evidence as candidate biomarkers of eCRS, particularly when measured in local nasal specimens.

Importantly, within two studies, eosinophilic inflammation was also identified in a subset of patients with CRSsNP: one reported significantly elevated ECP levels compared with non-Type 2 CRSsNP and healthy controls, while another found no significant difference in EPX levels between eosinophilic CRSwNP and eosinophilic CRSsNP, suggesting that an eosinophilic endotype may exist independently of nasal polyp phenotype [24,36]. These findings support the concept that inflammatory endotype is not fully captured by nasal-polyp status. Biomarker-based endotyping may therefore identify type 2 inflammation in patients who would otherwise be classified primarily by phenotype, although the therapeutic implications for CRSsNP require prospective study.

### 4.2. Specimen Source: A Reliability Gradient

One of the more clinically important patterns to emerge from this review concerns the specimen type used to measure eosinophil granule proteins. Tissue sampling produced the strongest discriminatory signal across biomarkers, including the highest AUC observed in this review (0.938 for tissue EPX) [36], but its need for biopsy or surgical access limits its practicality for serial or outpatient monitoring.

Nasal secretions/mucus are a promising minimally invasive alternative, with nasal-secretion ECP and EPX discriminating eosinophilic from non-eosinophilic CRS with AUCs of 0.82–0.90 and sensitivity/specificity in the 70–93% range [22,37,38,66].

Peripheral blood measurements, by contrast, were considerably more variable and represent an important pattern identified in this review. At least five independent studies reported serum or unspecified peripheral-blood ECP findings that were null in cohorts where tissue or nasal-secretion ECP from the same or comparable patients was clearly and significantly elevated [25,29,42,54,83]. This is a recurring discrepancy where we see a positive local (mucosal) signal alongside a null systemic (blood) signal for the same biomarker, suggesting that serum and blood granule protein levels are a less reliable surrogate for local mucosal eosinophilic inflammation than nasal secretion or tissue sampling.

These findings suggest a specimen-dependent gradient in diagnostic reliability: tissue provides the strongest signal but is the most invasive; nasal secretions offer diagnostic accuracy that approaches tissue sampling while remaining minimally invasive; and peripheral blood measurements, while the most accessible, are the least reliable, with multiple studies reporting non-significant associations even when the same biomarker was informative in tissue or local secretions.

### 4.3. Objective Severity Measures vs. Patient-Reported Outcomes

Granule proteins, including most consistently ECP and EPX, tracked objective radiographic (Lund-Mackay CT score) and endoscopic (Lund-Kennedy) severity measures more reliably than patient-reported outcome measures such as SNOT-22 and visual analogue scale (VAS) symptom scores. ECP correlated significantly with CT scores across multiple cohorts [40,67,75], while tissue MBP, tissue EPX, and mucus EDN were also associated with greater radiographic disease burden [34,36,39,74]. However, null associations between these proteins and CT scores were also reported [19,42,46,52,68,69]. Associations with patient-reported outcome measures were similarly heterogeneous [22,23,26,32,34,36,38,40,52,57,66,69,70,73,74,84,85]. Thus, granule proteins may reflect mucosal inflammatory activity more reliably than patients’ perceived symptom burden and should complement, rather than replace, patient-reported outcomes.

Granule protein levels did not consistently parallel nasal polyp burden. Two studies found significant associations between serum EDN and serum ECP and nasal polyp scores [71,80], while another study found that ECP was associated with asthma but not NPS [19]. These findings suggest that nasal polyp burden may be an imperfect representation of the degree of underlying Type 2 inflammation.

### 4.4. Interpretation of Prognostic Utility

Eosinophil granule proteins, and ECP in particular, showed reproducible prognostic value for postoperative recurrence across independent cohorts, whether measured in tissue, serum, or nasal secretions [20,21,40,44,46,67,84]; note that [20,21] derive from the same investigative group and overlapping cohort, so this should not be read as seven fully independent replications. Composite risk models incorporating ECP alongside IL-5, anti-dsDNA IgG, asthma status, and radiographic severity meaningfully improved predictive performance over clinical variables alone, and stepwise increases in tissue ECP across low-, intermediate-, and high-risk recurrence groups, along with a nearly ten-fold hazard ratio for recurrence in high-risk versus low-risk patients, were reproduced when the cohort was extended with an additional validation set [20,21]. This supports ECP-inclusive composite risk scores as a beneficial tool for identifying patients at an increased risk of recurrence who might benefit from closer postoperative surveillance or earlier consideration of adjunctive medical therapy. Notably, one study found that ECP measured in uncinate tissue was a substantially stronger predictor of recurrence than ECP measured in nasal polyp tissue itself [75], highlighting that biopsy location may meaningfully affect the prognostic utility of tissue-based eosinophil biomarkers. EDN was also associated with recurrence within a lipid-mediator and IL-13 inflammatory signature [49]. However, a few studies showed no associations of serum ECP, tissue ECP, and tissue MBP with recurrence risk [42,68,83], underscoring the need for standardized sampling and prospective validation.

Evidence for granule proteins as predictors of revision ESS was more limited. ECP and EPX were associated with recurrent or postoperative disease burden but did not consistently predict revision surgery [24,47,60]. While this is an important distinction to make, it is also important to note that the decision to undergo revision surgery is not only driven by the extent of inflammatory disease, but by symptoms, patient preference, practice patterns, and other factors.

Two studies suggest that eosinophilic inflammation can persist despite apparent clinical and surgical success. One found that ECP levels remained unchanged after ESS despite significant improvements in symptoms, radiographic findings, and other inflammatory markers [32], while another reported persistently elevated serum ECP in patients with residual postoperative inflammation [80]. Together, these findings suggest that eosinophil granule proteins may detect persistent subclinical inflammation not captured by conventional clinical or radiographic assessment, which could have implications in biomarker-guided postoperative monitoring despite symptom improvement.

Finally, EDN and ECP were associated with clinical disease control status. Mucus EDN was an independent predictor of uncontrolled CRS with good discriminatory ability [34,85], and serum ECP predicted postoperative uncontrolled disease specifically within the Type 2 endotype, with no predictive value in non-Type 2 disease [70]. Tissue ECP followed the same pattern, with eCRSwNP showing poorer one-year postoperative disease control than non-eCRSwNP or CRSsNP [78]. These findings suggest that the prognostic value of CRS disease control, as assessed by eosinophil granule proteins, is endotype-dependent and should therefore be evaluated in Type 2 and non-Type 2 CRS populations.

### 4.5. Practical Considerations

Beyond diagnostic and prognostic performance, clinical utility depends on practical considerations. For example, tissue sampling provides a direct assessment of sinonasal inflammation but requires biopsy, whereas nasal secretion or swab-based sampling may be less invasive, and blood-based testing is more readily accessible. Heterogeneity in specimen collection and assay methods, along with the lack of externally validated diagnostic thresholds, currently limits clinical implementation. Standardized assays and independently validated thresholds are thus needed to determine whether these biomarkers provide clinically meaningful information beyond established CRS phenotype measures, especially since no study reported cost, turnaround time, or reproducibility, which would be needed to demonstrate a benefit of any new assay over peripheral blood eosinophil count.

### 4.6. Clinical and Therapeutic Implications in the Era of Biologic Therapy

The development of biologic therapies targeting type 2 inflammation has transformed the management of severe or refractory CRSwNP. Currently available biologics include dupilumab, which blocks IL-4/IL-13 signaling and thereby dampens Type 2 cytokine effects on epithelial and immune cells; mepolizumab, which inhibits IL-5 and thereby reduces eosinophil maturation and survival; and omalizumab, which targets circulating IgE to attenuate allergic inflammation [88]. By interrupting these distinct components of the Type 2 inflammatory cascade, these therapies achieve their greatest clinical benefit in patients with genuine Type 2 eosinophilic inflammation, underscoring the importance of accurate endotyping before treatment selection.

As biologics become increasingly integrated into CRS management, there is a growing need for reliable, minimally invasive biomarkers for two distinct roles: to identify patients with active Type 2 inflammation and track response to treatment. The findings of this review directly support the idea that diagnostic biomarkers identify type 2 disease at baseline and are useful for monitoring clinical improvement with treatment. However, identifying patients who will derive differential benefit from a given therapy requires evidence that the biomarker status modifies the treatment effect, which was not well reported in the studies included in this review. In one included study of biologic therapy, EPX tracked treatment response longitudinally, falling significantly after both endoscopic sinus surgery and dupilumab therapy, with an AUC of 0.93 for a clinically meaningful symptomatic improvement and higher baseline EPX predicting greater improvement with dupilumab specifically [23]. This raises the possibility that EPX (and potentially other granule proteins) could serve both as a diagnostic tool for identifying Type 2-driven disease at baseline, and as a biomarker for monitoring response to biologic therapy. This is consistent with emerging evidence from one study outside the scope of our formal inclusion criteria in which Sarnoch et al. reported that EPX declines following dupilumab therapy and may serve as an objective indicator of treatment response, although more prospective studies are needed to validate its routine clinical use [89]. Because most approved biologics target a type 2 inflammatory pathway, an endotype biomarker at baseline informs whether type 2 inflammation is present rather than which biologic agent to choose from. Prospective studies comparing biomarker-defined subgroups across treatments are required before any granule protein can be described as a predictive biomarker. Given the substantial cost and clinical restrictions associated with biologic therapy, validated biomarkers capable of identifying appropriate candidates and monitoring treatment response could substantially improve precision medicine in CRS, and represent, in our view, one of the most promising translational applications of the evidence synthesized in this review.

### 4.7. Limitations

This review has several limitations that should be addressed. The first is that the substantial heterogeneity across included studies in biomarker assay methods, specimen sources, disease definitions, and outcome measures precluded quantitative meta-analysis. Because of this, findings were synthesized narratively, which inherently limits comparison of absolute concentrations and proposed cutoffs. Narrative synthesis may also obscure smaller or inconsistent effects. This review was restricted to English-language, peer-reviewed studies published after 2000, which may have excluded relevant non-English literature and introduces the potential for publication bias toward statistically significant, positive findings. Multiple publications come from the same investigative groups, some possibly drawing on overlapping cohorts, meaning the 71 included publications likely represent fewer unique populations, which may overstate the consistency of findings. Finally, eligible studies were required to report a predefined clinical outcome; therefore, studies that may have evaluated granule proteins mechanistically without clinical correlation were not included, which may understate the broader mechanistic literature on eosinophil degranulation in CRS. These limitations, together with the predominance of retrospective, low sample size, and/or single-center studies within our study cohort, support the need for prospective, multicenter validation using standardized specimen collection, assays, and outcome definitions.

## 5. Conclusions

Eosinophil granule proteins demonstrate promise as both diagnostic and prognostic biomarkers of eosinophilic CRS. Nasal secretion- and tissue-based sampling outperform serum measurements of the same biomarkers, supporting a shift toward minimally invasive, mucosal-compartment assays for both initial disease endotyping and longitudinal monitoring. These biomarkers track objective radiographic and endoscopic disease burden more reliably than subjective patient-reported symptom scores, indicating that they capture a distinct, though clinically important, dimension of disease activity. As biologic therapies targeting Type 2 inflammation become increasingly central to CRS management, minimally invasive eosinophil granule protein assays may offer a promising non-surgical strategy for identifying appropriate candidates for biologic therapy and for monitoring treatment response over time. Ultimately, incorporating these minimally invasive biomarkers into routine clinical practice may help clinicians move beyond phenotype-based classification toward endotype-driven, individualized management of chronic rhinosinusitis. Prospective studies with standardized assays and validated thresholds will be needed to confirm this potential.

## Figures and Tables

**Table 1:** Characteristics of included studies.

Reference	Year	Study Design	Risk of Bias (NOS)	Study Population (N; Phenotype/Endotype)	Biomarker(s)	Specimen Source	Comorbidities Assessed	Outcome(s) Assessed
Arjomandi et al. [16]	2013	Cross-sectional	Moderate	*n* = 29; Mixed CRS (CRSwNP and CRSsNP)	MBP	Tissue	NR	Phenotyping; Severity correlation
Bachert C et al. [17]	2000	Cross-sectional	Moderate	*n* = 31; CRSwNP	ECP	Tissue	Asthma, AR/Atopy	Phenotyping; Treatment response
Bachert C et al. [18]	2001	Cross-sectional	Moderate	*n* = 40; CRSwNP	ECP	Tissue	Asthma, ASA sensitivity, AR/Atopy	Phenotyping
Bachert C et al. [19]	2010	Cross-sectional	Moderate	*n* = 163; CRSwNP	ECP	Tissue; Serum/Plasma	Asthma, AR/Atopy	Phenotyping; Severity correlation; Clinical outcome
Bai J et al. [20]	2022	Cohort (prospective)	Moderate	*n* = 94; CRSwNP	ECP	Tissue	Asthma, AERD, AR/Atopy	Phenotyping; Severity correlation; Recurrence/Prognosis
Bai J et al. [21]	2026	Cohort (prospective)	Low	*n* = 155; CRSwNP	ECP	Tissue	Asthma, AERD, AR/Atopy	Phenotyping; Severity correlation; Recurrence/Prognosis
Callander J et al. [22]	2025	Cross-sectional	Moderate	*n* = 42; eCRS, non-eCRS	EPX	Nasal secretion/mucus; Nasal swab/brush	Asthma, ASA sensitivity, AR/Atopy	Endotyping; Severity correlation; Clinical outcome
Callander J et al. [23]	2026	Cohort (prospective)	Moderate	*n* = 36; eCRS, non-eCRS	EPX	Nasal secretion/mucus; Nasal swab/brush	Asthma, AR/Atopy	Endotyping; Severity correlation; Treatment response
Delemarre et al. [24]	2020	Cohort (retrospective)	Low	*n* = 240; CRSsNP (Type 2 vs. non-Type 2)	ECP	Tissue	Asthma, AR/Atopy	Endotyping; Phenotyping; Severity correlation; Recurrence/Prognosis
Di Lorenzo et al. [25]	2001	Cross-sectional	Moderate	*n* = 87; CRSwNP	ECP	Serum/Plasma; Lavage/wash	AR/Atopy	Phenotyping; Severity correlation
Dimić et al. [26]	2022	Cross-sectional	Moderate	*n* = 90; NP categorized into NANP and ANP	ECP	Nasal secretion/mucus	AR/Atopy	Phenotyping; Severity correlation
Divekar R et al. [27]	2017	Cross-sectional	Moderate	*n* = 32; CRSwNP, CRSsNP	MBP	Tissue	Asthma	Phenotyping; Severity correlation
Divekar R et al. [28]	2015	Cohort (prospective)	Moderate	*n* = 45; CRSwNP	MBP	Serum/Plasma; Nasal secretion/mucus; Lavage/wash	Asthma, ASA sensitivity, AERD, AR/Atopy	Phenotyping; Severity correlation; Disease control
Dupuch et al. [29]	2018	Cross-sectional	Moderate	*n* = 29; CRSwNP	ECP	Peripheral blood	Asthma, ASA sensitivity, AERD, AR/Atopy	Phenotyping; Severity correlation; Clinical outcome
Ediger et al. [30]	2005	Cross-sectional	Moderate	*n* = 17; NP	EPX	Tissue; Serum/Plasma	Asthma, ASA sensitivity, AERD, AR/Atopy	Phenotyping; Severity correlation
Gröger et al. [31]	2013	Cross-sectional	Moderate	*n* = 120; CRS, NP, AR	ECP	Tissue; Nasal swab/brush; Peripheral blood	AR/Atopy	Phenotyping; Clinical outcome
Harmon et al. [32]	2024	Cohort (prospective)	Low	*n* = 206; CRSsNP, CRSwNP, Type 2-high vs. Type 2-low	ECP	Nasal secretion/mucus	Asthma, ASA sensitivity	Endotyping; Phenotyping; Severity correlation; Recurrence/Prognosis
Hsu et al. [33]	2026	Case–control	Low	*n* = 86; CRSwNP (CCAD vs. non-CCAD)	ECP	Tissue; Peripheral blood	Asthma, AR/Atopy	Phenotyping; Severity correlation; Treatment response
Huang X et al. [34]	2024	Cross-sectional	Low	*n* = 175; CRSwNP, CRSsNP, eCRS, non-eCRS, controlled CRS, uncontrolled CRS	EDN	Tissue; Nasal secretion/mucus; Peripheral blood	Asthma, AR/Atopy	Endotyping; Phenotyping; Severity correlation; Disease control
Huang Z et al. [35]	2024	Cross-sectional	Moderate	*n* = eCRSwNP non-eCRSwNP	ECP	Tissue	NR	Endotyping
Idler et al. [36]	2024	Cross-sectional	Moderate	*n* = 28; CRSwNP, CRSsNP, eCRS, neCRS, eCRSsNP, eCRSwNP	EPX	Tissue	Asthma, AERD, AR/Atopy	Endotyping; Phenotyping; Severity correlation; Clinical outcome
Jin J et al. [37]	2023	Cross-sectional	Moderate	*n* = 92; CRSwNP, eCRS, non-eCRS	ECP	Tissue; Nasal secretion/mucus	AR/Atopy	Endotyping; Phenotyping; Severity correlation
Jin J et al. [38]	2023	Cross-sectional	Moderate	*n* = 139; CRSwNP, CRSsNP	ECP	Tissue; Nasal secretion/mucus; Peripheral blood	AR/Atopy	Phenotyping; Severity correlation
Kim D et al. [39]	2014	Cross-sectional	Moderate	*n* = 119; CRSwNP, CRSsNP, eCRSwNP, non-eCRSwNP	MBP	Tissue	AR/Atopy	Endotyping; Phenotyping; Severity correlation
Kim K et al. [40]	2013	Cohort (retrospective)	Low	*n* = 492; CRS without AR, CRS with AR	ECP	Serum/Plasma; Nasal secretion/mucus	AR/Atopy	Phenotyping; Severity correlation; Recurrence/Prognosis
Lal et al. [41]	2019	Cross-sectional	Moderate	*n* = 101; CRSwNP, CRSsNP	EPX	Tissue	Asthma, ASA sensitivity, AR/Atopy	Phenotyping; Severity correlation
Li Q et al. [42]	2016	Cross-sectional	Moderate	*n* = 210; CRSwNP, atopic vs. non-atopic	ECP	Serum/Plasma	AR/Atopy	Phenotyping; Severity correlation; Recurrence/Prognosis
Li X et al. [43]	2019	Cross-sectional	Moderate	*n* = 157; CRSwNP, CRSsNP, eCRS, non-eCRS	ECP	Tissue	NR	Endotyping; Phenotyping; Severity correlation
Lin L et al. [44]	2024	Cohort (prospective)	Low	*n* = 200; eCRSwNP	ECP	Tissue; Serum/Plasma	AR/Atopy	Endotyping; Severity correlation; Recurrence/Prognosis
Lin Y et al. [45]	2021	Cross-sectional	Moderate	*n* = 67; Central-compartment CRS vs. other CRS (CRSwNP, CRSsNP, eCRS)	ECP	Tissue; Peripheral blood	Asthma, AR/Atopy	Endotyping; Phenotyping; Severity correlation
Lu et al. [46]	2021	Cohort (prospective)	Low	*n* = 58; CRSwNP	ECP	Serum/Plasma	Asthma, AR/Atopy	Phenotyping; Severity correlation; Recurrence/Prognosis
Massey et al. [47]	2024	Cohort (prospective)	Moderate	*n* = 47; CRSwNP, CRSsNP, eCRS, non-eCRS	EPX	Tissue; Nasal swab/brush	Asthma, AERD, AR/Atopy	Endotyping; Phenotyping; Severity correlation; Recurrence/Prognosis; Treatment response; Disease control
Matsuwaki et al. [48]	2013	Cross-sectional	Moderate	*n* = 45; CRSwNP, eCRS, non-eCRS, AFRS	ECP	Tissue; Serum/Plasma	Asthma, AR/Atopy	Endotyping; Phenotyping; Severity correlation
Nordström et al. [49]	2024	Cohort (prospective)	Low	*n* = 66; CRSwNP	EDN	Tissue; Nasal secretion/mucus	Asthma, AERD, AR/Atopy	Phenotyping; Severity correlation; Clinical outcome
Pérez-Novo C et al. [50]	2004	Cross-sectional	Moderate	*n* = 52; CRSwNP (aspirin sensitive vs. aspirin tolerant)	ECP	Tissue	Asthma, ASA sensitivity, AERD, AR/Atopy	Phenotyping
Pérez-Novo C et al. [51]	2005	Cross-sectional	Moderate	*n* = 45; CRSsNP, CRSwNP, Aspirin-sensitive CRSwNP (CRS-ASNP)	ECP	Tissue	Asthma, AERD, AR/Atopy	Phenotyping
Perić A et al. [52]	2023	Cross-sectional	Low	*n* = 90; CRSwNP, AERD	ECP	Nasal secretion/mucus	Asthma, ASA sensitivity	Phenotyping; Severity correlation
Perić A et al. [53]	2011	Cross-sectional	Moderate	*n* = 42; Atopic vs. non-atopic CRSwNP	ECP	Tissue; Nasal secretion/mucus	Asthma, ASA sensitivity, AERD, AR/Atopy	Phenotyping
Phetpong et al. [54]	2022	Randomized controlled trial	Low	*n* = 20; CRSwNP	ECP	Serum/Plasma; Nasal secretion/mucus	NR	Phenotyping
Ponikau J et al. [55]	2005	Cross-sectional	Moderate	*n* = 34; CRS	MBP	Tissue; Nasal secretion/mucus	Asthma, ASA sensitivity, AR/Atopy	Eosinophilic inflammation
Ponikau J et al. [56]	2015	Cross-sectional	Moderate	*n* = 103; CRS	MBP	Nasal secretion/mucus	AR/Atopy	Eosinophilic inflammation
Racette et al. [57]	2017	Cross-sectional	Moderate	*n* = 258; post-surgical CRSwNP	EDN, MBP	Nasal secretion/mucus	NR	Severity correlation; Clinical outcome
Rank et al. [58]	2013	Cohort (prospective)	Moderate	*n* = 9; CRSwNP	EDN, MBP	Nasal secretion/mucus; Lavage/wash	Asthma, AERD, AR/Atopy	Phenotyping; Severity correlation; Disease control
Rudack et al. [59]	2004	Cross-sectional	Moderate	*n* = 78; CRSwNP	ECP	Tissue	Asthma, ASA sensitivity, AR/Atopy	Phenotyping
Ryu et al. [60]	2019	Cross-sectional	Moderate	*n* = 179; CRSwNP, primary NP, refractory NP, eosinophilic NP, non-eosinophilic NP	ECP	Tissue; Serum/Plasma	Asthma, AR/Atopy	Endotyping; Phenotyping; Severity correlation
Sabirov et al. [61]	2008	Cross-sectional	Moderate	*n* = 42; CRSwNP vs. CRSsNP	ECP	Tissue; Serum/Plasma	Asthma, AR/Atopy	Phenotyping
Scavuzzo et al. [62]	2005	Cross-sectional	Moderate	*n* = 44; CRSwNP (atopic vs. non-atopic)	ECP	Nasal secretion/mucus	AERD, AR/Atopy	Phenotyping
Schmid et al. [63]	2010	Cross-sectional	Moderate	*n* = 44; CRSwNP	MBP	Nasal secretion/mucus	AR/Atopy	Phenotyping; Severity correlation
Shi et al. [64]	2013	Cross-sectional	Moderate	*n* = 375; CRSsNP, eCRSwNP, non-eCRSwNP	ECP	Tissue	NR	Endotyping; Severity correlation
Sit et al. [65]	2023	Cross-sectional	Moderate	*n* = 38; Primary diffuse type 2 CRS (CCAD vs. eCRS)	EPX	Tissue; Peripheral blood	AR/Atopy	Endotyping; Phenotyping
Smith et al. [66]	2023	Cross-sectional	Moderate	*n* = 54; CRSwNP, CRSsNP, eCRS, non-eCRS	EPX	Tissue; Nasal swab/brush	Asthma, AERD, AR/Atopy	Endotyping; Phenotyping, Severity correlation
Stevens et al. [14]	2015	Cross-sectional	Moderate	*n* = 56.; CRSsNP, CRSwNP, AERD	ECP	Tissue; Lavage/wash	Asthma, AERD, AR/Atopy	Phenotyping; Recurrence/Prognosis
Sun et al. [67]	2009	Cohort (prospective)	Low	*n* = 98; CSRwNP	ECP	Tissue; Serum/Plasma; Nasal secretion/mucus	Asthma, ASA sensitivity, AERD, AR/Atopy	Phenotyping; Severity correlation; Recurrence/Prognosis
Suzuki et al. [68]	2022	Cross-sectional	High	*n* = 128; eCRS, probable eCRS, probable non-eCRS, non-eCRS	MBP	Tissue	Asthma, AR/Atopy	Endotyping; Severity correlation; Recurrence/Prognosis
Topal et al. [69]	2014	Cross-sectional	Moderate	*n* = 44; CRSwNP	ECP	Lavage/wash	Asthma, AERD, AR/Atopy	Phenotyping; Severity correlation
Tsai et al. [70]	2024	Cohort (prospective)	Low	*n* = 113; CRSwNP, CRSsNP, T2 CRS, non-T2 CRS	ECP	Serum/Plasma	Asthma, AR/Atopy	Endotyping; Phenotyping; Severity correlation; Disease control
Tsuda et al. [71]	2019	Cross-sectional	Moderate	*n* = 123; ECRS vs. NECRS (CRSwNP subtype)	EDN	Tissue; Serum/Plasma	AR/Atopy	Endotyping; Severity correlation
Vagic et al. [72]	2008	Cross-sectional	Moderate	*n* = 53; CRSsNP	ECP	Tissue; Lavage/wash	Asthma, AR/Atopy	Phenotyping
Van Zele et al. [73]	2006	Cross-sectional	Moderate	*n* = 40; CRSsNP, CRSwNP, CF-associated nasal polyps, Th1 vs. Th2	ECP	Tissue	Asthma, ASA sensitivity, AR/Atopy	Endotyping; Phenotyping; Severity correlation
Vukadinović et al. [74]	2024	Cross-sectional	Moderate	*n* = 60; CRSwNP	MBP	Tissue	Asthma, ASA sensitivity, AERD	Phenotyping; Severity correlation
Weibman et al. [75]	2017	Cohort (prospective)	Low	*n* = 116; CRSwNP	ECP	Tissue	Asthma, AERD, AR/Atopy	Phenotyping; Severity correlation; Recurrence/Prognosis
Wu C et al. [76]	2012	Cross-sectional	Moderate	*n* = 40; CRSwNP	MBP	Tissue	Asthma, ASA sensitivity, AERD, AR/Atopy	Phenotyping; Severity correlation
Wu D et al. [77]	2023	Cross-sectional	Low	*n* = 175; CRSwNP, CRSsNP, eCRS, non-eCRS	EDN	Tissue; Nasal secretion/mucus	Asthma, AERD, AR/Atopy	Endotyping; Severity correlation; Clinical outcome
Wu M et al. [78]	2025	Cross-sectional	Moderate	*n* = 86; CRSsNP, nECRSwNP, ECRSwNP	ECP	Tissue	NR	Endotyping; Severity correlation; Disease control
Wu P et al. [79]	2025	Cross-sectional	Low	*n* = 170; CRSwNP	ECP	Tissue; Serum/Plasma	Asthma, AR/Atopy	Phenotyping; Severity correlation
Wu P et al. [80]	2026	Cohort (prospective)	Low	*n* = 143; CRSwNP	ECP	Tissue; Serum/Plasma	Asthma, ASA sensitivity, AR/Atopy	Phenotyping; Severity correlation; Recurrence/Prognosis
Yao et al. [81]	2024	Cross-sectional	Moderate	*n* = 48; CRSwNP	EPX, ECP	Lavage/wash; Sputum	Asthma	Phenotyping; Severity correlation
Yun et al. [82]	2020	Cross-sectional	Moderate	*n* = 20; eCRS vs. non-eCRS	MBP, ECP, EDN, EPX	Tissue; Peripheral blood	Asthma	Endotyping; Severity correlation; Clinical outcome
Zhang N et al. [83]	2006	Cross-sectional	Moderate	*n* = 47; CRSwNP	ECP	Tissue; Serum/Plasma	Asthma, AR/Atopy	Phenotyping
Zhang Q et al. [84]	2024	Cohort (retrospective)	Moderate	*n* = 226; CRSwNP vs. CRSsNP	ECP	Serum/Plasma	Asthma, ASA sensitivity	Phenotyping; Severity correlation; Recurrence/Prognosis
Zhang Y et al. [85]	2025	Cross-sectional	Moderate	*n* = 150; CRS (uncontrolled vs. controlled)	EDN	Nasal secretion/mucus; Peripheral blood	Asthma, AERD, AR/Atopy	Phenotyping; Severity correlation; Disease control

Abbreviations: CRS, chronic rhinosinusitis; CRSwNP, chronic rhinosinusitis with nasal polyps; CRSsNP, chronic rhinosinusitis without nasal polyps; eCRS/ECRS, eosinophilic chronic rhinosinusitis; neCRS/NECRS/non-eCRS, non-eosinophilic chronic rhinosinusitis; eCRSwNP/ ECRSwNP, eosinophilic chronic rhinosinusitis with nasal polyps; eCRSsNP, eosinophilic chronic rhinosinusitis without nasal polyps; non-eCRSwNP/ nECRSwNP, non-eosinophilic chronic rhinosinusitis with nasal polyps; ECP, eosinophil cationic protein; MBP, major basic protein; EPX, eosinophil peroxidase; EDN, eosinophil-derived neurotoxin; AR, allergic rhinitis; AERD, aspirin-exacerbated respiratory disease; ASA, acetylsalicylic acid; AFRS, allergic fungal rhinosinusitis; CCAD, central compartment atopic disease; CF, cystic fibrosis; Th1, T helper 1; Th2, T helper 2; T2, type 2; NOS, Newcastle–Ottawa Scale; *n*, number of participants; NR, not reported.

**Table 2:** Eosinophil Granule Protein Biomarker Frequency. This table demonstrates the frequency with which each of the eosinophil granule proteins were evaluated in the studies meeting inclusion criteria for this systematic review.

Biomarker	Studies (*n*)	% of Included Studies (*n* = 71)
ECP	46	65%
MBP	13	18%
EPX	10	14%
EDN	8	11%

Abbreviations: ECP, eosinophil cationic protein; MBP, major basic protein; EPX, eosinophil peroxidase; EDN, eosinophil-derived neurotoxin; *n*, number of studies.

**Table 3:** Specimen Source Frequency. This table shows the specimen source from which eosinophil granule proteins were evaluated across all of the studies meeting inclusion criteria for this systematic review.

Specimen Source	Studies (*n*)	% of Included Studies (*n* = 71)
Tissue	48	68%
Nasal secretions/mucus	30	42%
Serum/plasma/peripheral blood	29	41%

**Table 4:** Diagnostic Performance of Eosinophil Granule Proteins in Chronic Rhinosinusitis.

Study	Biomarker (Specimen)	Outcome	Effect Size (OR/HR, 95% CI)	*p*-Value
Jin et al. [37]	ECP (nasal secretion)	eCRS vs. non-eCRS; tissue eosinophilia reference standard	AUC 0.823; cutoff 73.88 μg/mL; sensitivity 85.4%; specificity 70.3%; threshold derived within cohort; external validation not reported.	<0.0001
Jin et al. [38]	ECP (nasal secretion)	Eos + Neu − CRS vs. Eos − Neu − CRS; endotype classification based on tissue eosinophilia	Cutoff 44.55 μg/mL; AUC 0.8801; sensitivity 87.9%; specificity 76.5%. Threshold derived within cohort; external validation not reported.	NR
Callander et al. [22]	EPX (nasal secretion)	eCRS vs. non-eCRS; histologic eosinophilia > 10 eos/HPF	Cutoff 118.35 ng/mL; AUC 0.89; sensitivity 71.4%; specificity 92.9%. Threshold derived within cohort; external validation not reported.	0.001
Idler et al. [36]	EPX (tissue)	eCRS vs. non-eCRS; tissue eosinophilia > 10 eos/HPF	Cutoff 470 ng/mL; AUC 0.938 (95% CI 0.835–1.00); sensitivity 81.3%; specificity 100%. Threshold derived within cohort; external validation not reported.	0.002
Smith et al. [66]	EPX (nasal swab)	eCRS vs. non-eCRS; histologic eosinophilia ≥ 10 eos/HPF	AUC 0.863; sensitivity 85.7%; specificity 79.0%; PPV 88.2%; NPV 75.0%; diagnostic accuracy 83.3%. Threshold derived within cohort; external validation not reported.	<0.0001

Abbreviations: ECP, eosinophil cationic protein; EPX, eosinophil peroxidase; eCRS, eosinophilic chronic rhinosinusitis; CRS, chronic rhinosinusitis; Eos, eosinophils; Neu, neutrophils; HPF, high-power field; AUC, area under the curve; CI, confidence interval; PPV, positive predictive value; NPV, negative predictive value; NR, not reported.

**Table 5:** Prognostic Utility of Eosinophil Granule Proteins: Disease Recurrence, Revision ESS, Persistent Inflammation, and Disease Control Status Biomarker.

Study	Biomarker (Specimen)	Outcome	Effect Size (OR/HR, 95% CI)	*p*-Value
**Disease Recurrence**				
Bai et al. [20]	ECP (nasal polyp tissue)	Polyp recurrence 2–5 years after ESS	Combined model AUC = 0.89 (vs. 0.78 biomarker-only, 0.79 clinical-only); ECP independently predictive on multivariable logistic regression	<0.05
Bai et al. [21]	ECP (nasal polyp tissue)	Postoperative polyp recurrence after ESS (up to 60 months follow-up)	HR: 9.8 (high-risk vs. low-risk); HR: 4.4 (intermediate vs. low-risk); AUC = 0.89 training/0.76 validation	<0.0001
Delemarre et al. [24]	ECP (sinonasal tissue)	Recurrence, Type 2 vs. non-Type 2 CRSsNP	19.0% (Type 2) vs. 9.2% (non-Type 2); OR/HR NR	<0.05
Kim et al. [40]	ECP (serum)	CRS recurrence after ESS	ECP 32.8 ± 20.4 vs. 19.8 ± 10.0 µg/L, recurrence vs. non-recurrence; OR/HR NR	0.036
Li et al. [42]	ECP (serum)	Recurrence, atopic vs. non-atopic CRSwNP	13/99 (13%) atopic vs. 9/111 (8%) non-atopic	NS
Lin et al. [44]	ECP (serum)	Postoperative recurrence at 6 months after ESS	OR: 1.283 (95% CI: 1.044–1.576); ROC AUC = 0.680	0.018
Lu et al. [46]	ECP (serum)	Early postoperative recurrence after FESS	OR: 54.8 (95% CI: 2.3–1327.3); HR: 7.6 (95% CI: 1.6–36.8); AUC = 0.729, NPV 94.7%	0.014 (OR); 0.011 (HR)
Nordström et al. [49]	EDN (nasal polyp tissue/secretions)	Polyp recurrence within inflammatory cluster	Recurrence 62.5% (high-risk cluster) vs. 19% (reference cluster); cluster independently associated with recurrence on multivariable regression; OR/HR NR	0.02 (cluster); <0.001 (EDN)
Sun et al. [67]	ECP (nasal secretion)	Postoperative polyp recurrence	ECP 287.7 vs. 131.9 ng/mL, recurrence vs. non-recurrence; OR/HR NR	<0.001
Suzuki et al. [68]	MBP (nasal polyp/ethmoid tissue)	Postoperative recurrence	MBP score not associated with recurrence; OR/HR NR	0.467
Weibman et al. [75]	ECP (uncinate tissue vs. nasal polyp tissue)	Polyp recurrence after ESS	Uncinate ECP ≥ 290 ng/mg: HR 2.84 (95% CI: 1.14–7.03); nasal polyp ECP ≥ 290 ng/mg: HR 1.74 (95% CI: 0.74–4.13)	0.025 (uncinate); 0.207 (nasal polyp)
Zhang Q et al. [84]	ECP (serum)	Postoperative recurrence after ESS	OR: 2.77; AUC = 0.77 alone, 0.84 with total IgE	0.003
**Revision ESS**				
Delemarre et al. [24]	ECP (sinonasal tissue)	Revision surgery among patients with recurrence	80.6% of recurrent patients required revision surgery; OR/HR NR	NR
Massey et al. [47]	EPX (intraoperative sinonasal swab)	Revision surgery after ESS	6/47 (13%); r = 0.17; OR/HR NR	0.24 (revision surgery)
Ryu et al. [60]	ECP (tissue and serum)	Refractory CRSwNP requiring revision ESS vs. primary disease	Serum ECP higher in refractory disease; tissue ECP NS; OR/HR NR	NS (tissue); 0.001 (serum)
Stevens et al. [14]	ECP (uncinate tissue, nasal polyp)	History of revision surgery, AERD vs. CRSwNP	Revision surgery more frequent in AERD; ECP not modeled as predictor	NR
**Persistent Inflammation**				
Harmon et al. [32]	ECP (middle meatal mucus)	ECP persistence after ESS despite symptomatic/radiographic improvement	ECP unchanged after ESS; OR/HR NR	0.15
Wu PW et al. [80]	ECP (serum)	Residual sinus inflammation 3 months after ESS (eCRSwNP)	ECP 62.7 vs. 38.4 µg/L; not independently predictive	0.039
**Disease Control Status**				
Divekar et al. [28]	MBP (nasal secretions)	Acute symptomatic exacerbation vs. baseline (CRSwNP)	Nasal MBP increased during exacerbation; OR/HR NR	0.02
Huang et al. [34]	EDN (nasal mucus)	Uncontrolled vs. controlled CRS	OR 1.323 (95% CI 1.175–1.659); AUC 0.897; sensitivity 92.3%, specificity 91.7%	0.004
Rank et al. [58]	MBP, EDN (nasal secretions/wash fluid)	Acute CRS exacerbation vs. baseline	MBP and EDN increased during exacerbation; OR/HR NR	<0.05
Tsai et al. [70]	ECP (serum)	Postoperative uncontrolled disease (Type 2 CRS)	OR 16.7 (95% CI 1.7–161.6); HR 11.3 (95% CI 1.5–87.3); AUC 0.715; NPV 96.8%	0.015 (OR); 0.020 (HR)
Wu M et al. [78]	ECP (ethmoid sinus tissue)	1-year postoperative disease control (eCRSwNP vs. non-eCRSwNP vs. CRSsNP)	Uncontrolled: 93% eCRSwNP vs. 27% non-eCRSwNP vs. 0% CRSsNP; OR/HR NR	Significant (exact value NR)
Zhang Y et al. [85]	EDN (nasal mucus)	Uncontrolled vs. controlled CRS	AUC 0.798 (95% CI 0.692–0.904); 425.36 vs. 214.73 pg/mL	<0.001

Abbreviations: ECP, eosinophil cationic protein; MBP, major basic protein; EPX, eosinophil peroxidase; EDN, eosinophil-derived neurotoxin; ESS, endoscopic sinus surgery; FESS, functional endoscopic sinus surgery; CRS, chronic rhinosinusitis; CRSwNP, chronic rhinosinusitis with nasal polyps; CRSsNP, chronic rhinosinusitis without nasal polyps; eCRS, eosinophilic chronic rhinosinusitis; eCRSwNP, eosinophilic chronic rhinosinusitis with nasal polyps; AERD, aspirin-exacerbated respiratory disease; OR, odds ratio; HR, hazard ratio; CI, confidence interval; AUC, area under the curve; ROC, receiver operating characteristic; NPV, negative predictive value; NS, not significant; NR, not reported; Type 2, type 2 inflammation; IgE, immunoglobulin E; r, correlation coefficient.

**Table 6:** Summary of Evidence for Eosinophil Granule Protein Biomarkers in Chronic Rhinosinusitis.

Biomarker	Studies *(n,* %)	Diagnostic Evidence	Severity Correlation	Prognostic Evidence	Key Gaps
ECP	46 (65%)	Most extensively studied; distinguishes eCRS/CRSwNP from comparators across multiple independent cohorts, although findings were not uniformly positive	Correlates with CT and endoscopy scores; less consistent with SNOT-22	Strongest prognostic evidence; independent predictor of postoperative recurrence and predicts postoperative uncontrolled Type 2 disease	No externally validated diagnostic threshold; systemic measurements less consistent than local/tissue secretions
MBP	13 (18%)	Consistently elevated in eCRS/CRSwNP, but mostly reported as a semi-quantitative staining/deposition scores rather than continuous concentrations	Correlates with CT/SNOT-22 in some but not all cohorts	Not associated with recurrence in the study evaluating this outcome; nasal MBP increased during acute exacerbations	Lack of standardized quantification limits diagnostic accuracy assessment
EPX	10 (14%)	Highest reported diagnostic accuracy: tissue AUC 0.938, nasal swab 0.89	Correlated with CT, SNOT-22, and/or Lund-Kennedy scores in individual cohorts	EPX changed following treatment, and higher baseline mucus EPX was associated with greater SNOT-22 improvement after dupilumab; not associated with revision surgery in one study	Few studies; no external threshold validation; limited evidence for postoperative outcomes
EDN	8 (11%)	Tissue EDN was associated with eosinophilic disease; nasal mucus EDN outperforms peripheral blood eosinophil counts for predicting disease control and olfactory dysfunction	Associated with CT, nasal polyp score, and JESREC score	Nasal mucus EDN independently predicted uncontrolled CRS and increased during acute exacerbations	No direct comparison with tissue eosinophil count as a reference standard; no external validation

Abbreviations: ECP, eosinophil cationic protein; MBP, major basic protein; EPX, eosinophil peroxidase; EDN, eosinophil-derived neurotoxin; eCRS, eosinophilic chronic rhinosinusitis; CRSwNP, chronic rhinosinusitis with nasal polyps; CT, computed tomography; SNOT-22, 22-item Sino-Nasal Outcome Test; AUC, area under the curve; JESREC, Japanese Epidemiological Survey of Refractory Eosinophilic Chronic Rhinosinusitis.

## Data Availability

No new data were created or analyzed in this study. Data sharing is not applicable to this article.

## Supplementary Materials

The following supporting information can be downloaded at https://www.mdpi.com/article/10.3390/jcm15207790/s1, Supplementary Material S1: Complete database-specific search strategies. This systematic review was conducted in accordance with the PRISMA 2020 guidelines, and the completed PRISMA 2020 checklist is provided in Supplementary Material S2.

## Abbreviations

The following abbreviations are used in this manuscript:

CRS	Chronic rhinosinusitis
eCRS	Eosinophilic chronic rhinosinusitis
Non-eCRS	Non-eosinophilic chronic rhinosinusitis
CRSwNP	Chronic rhinosinusitis with nasal polyps
CRSsNP	Chronic rhinosinusitis without nasal polyps
MBP	Major basic protein
ECP	Eosinophil cationic protein
EPX	Eosinophil peroxidase
EDN	Eosinophil-derived neurotoxin
EDGP	Eosinophil-derived granule protein
RNase 2	Ribonuclease 2 (EDN)
RNase 3	Ribonuclease 3 (ECP)
MPO	Myeloperoxidase
IgE	Immunoglobulin E
IL	Interleukin
IFN-γ	Interferon-gamma
TNF-α	Tumor necrosis factor-alpha
AERD	Aspirin-exacerbated respiratory disease
ASA	Aspirin sensitivity
CCAD	Central compartment atopic disease
AFRS	Allergic fungal rhinosinusitis
JESREC	Japanese Epidemiological Survey of Refractory Eosinophilic Chronic Rhinosinusitis
ESS	Endoscopic sinus surgery
FESS	Functional endoscopic sinus surgery
SNOT-22	22-item Sino-Nasal Outcome Test
SNOT-20	20-item Sino-Nasal Outcome Test
VAS	Visual analogue scale
TNSS	Total nasal symptom score
NPS	Nasal Polyp Score
CT	Computed tomography
hpf	High-power field
IHC	Immunohistochemistry
ELISA	Enzyme-linked immunosorbent assay
qRT-PCR	Quantitative reverse transcription polymerase chain reaction
AUC	Area under the curve
OR	Odds ratio
HR	Hazard ratio
CI	Confidence interval
NPV	Negative predictive value
PPV	Positive predictive value
LTE4	Leukotriene E4
PGD2	Prostaglandin D2
15(S)-HETE	15(S)-hydroxyeicosatetraenoic acid
PRISMA	Preferred Reporting Items for Systematic Reviews and Meta-Analyses
PROSPERO	International Prospective Register of Systematic Reviews
NOS	Newcastle-Ottawa Scale
GRADE	Grading of Recommendations Assessment, Development and Evaluation
RCT	Randomized controlled trial
