# Supplementary material for: Eosinophil Granule Proteins in Chronic Rhinosinusitis: A Systematic Review of Biomarker Utility for Disease Characterization and Outcomes
Source: J Clin Med. 2026 Oct 9;15(20):7790. doi: 10.3390/jcm15207790 (PMC13705240; doi:10.3390/jcm15207790)
Supplement: Supplementary file 1 [file jcm-15-07790-s001.zip › jcm-4586774-supplementary-S1-revised.pdf]

**Supplementary Material S1. Complete Database Search Strategies**

The following database-specific search strategies were used for the systematic review. Searches were conducted on June 12, 2026. The complete syntax, controlled vocabulary, field tags, truncation operators, and search results are provided below.

**Database:** PubMed (NCBI)

**Final search date:** June 12, 2026

**Results retrieved:** 276

**Search strategy:**

( "Sinusitis"[Mesh] OR "Nasal Polyps"[Mesh] OR "chronic rhinosinusitis"[tiab] OR "chronic sinusitis"[tiab] OR "rhinosinusitis"[tiab] OR "nasal polyp"[tiab] OR "nasal polyposis"[tiab] ) AND ( "eosinophil granule protein*"[tiab] OR "eosinophil cationic protein"[tiab] OR "RNase 3"[tiab] OR "eosinophil major basic protein"[tiab] OR "major basic protein"[tiab] OR "eosinophil-derived neurotoxin"[tiab] OR "eosinophil derived neurotoxin"[tiab] OR "RNase 2"[tiab] OR "eosinophil peroxidase"[tiab] )

**Database:** Web of Science

**Final search date:** June 12, 2026

**Results retrieved:** 92

**Search strategy:**

(sinusitis (Topic) or nasal polyp (Topic) or chronic rhinosinusitis (Topic) or chronic sinusitis (Topic) or rhinosinusitis (Topic) or nasal polyposis (Topic)) and (eosinophil granule protein (Topic) or eosinophil cationic protein (Topic) or RNase 3 (Topic) or eosinophil major basic protein (Topic) or major basic protein (Topic) or eosinophil-derived neurotoxin (Topic) or eosinophil derived neurotoxin (Topic) or RNase 2 (Topic) or eosinophil peroxidase (Topic))

**Database:** Cochrane Library

**Final search date:** June 12, 2026

**Results retrieved:** 18

**Search strategy:**

( [mh "Sinusitis"] OR [mh "Nasal Polyps"] OR "chronic rhinosinusitis":ti,ab,kw OR "chronic sinusitis":ti,ab,kw OR "rhinosinusitis":ti,ab,kw OR "nasal polyp":ti,ab,kw OR "nasal polyposis":ti,ab,kw ) AND ( [mh "Eosinophil Granule Proteins"] OR "eosinophil granule protein*":ti,ab,kw OR "eosinophil cationic protein":ti,ab,kw OR "RNase 3":ti,ab,kw OR "eosinophil major basic protein":ti,ab,kw OR

"major basic protein":ti,ab,kw OR "eosinophil-derived neurotoxin":ti,ab,kw OR "eosinophil derived neurotoxin":ti,ab,kw OR "RNase 2":ti,ab,kw OR "eosinophil peroxidase":ti,ab,kw )

Cochrane Advanced Search Criteria

| ID | Search | Hits |
|---|---|---|
| #1 | MeSH descriptor: [Sinusitis] explode all trees | 1604 |
| #2 | MeSH descriptor: [Nasal Polyps] explode all trees | 670 |
| #3 | (chronic rhinosinusitis):ti,ab,kw | 1882 |
| #4 | (chronic sinusitis):ti,ab,kw | 1872 |
| #5 | (rhinosinusitis):ti,ab,kw | 2265 |
| #6 | (nasal polyp):ti,ab,kw | 978 |
| #7 | (nasal polyposis):ti,ab,kw | 636 |
| #8 | #1 OR #2 OR #3 OR #4 OR #5 OR #6 OR #7 | 4077 |
| #9 | MeSH descriptor: [Eosinophil Granule Proteins] explode all trees | 321 |
| #10 | (eosinophil granule protein):ti,ab,kw | 185 |
| #11 | (eosinophil cationic protein):ti,ab,kw | 571 |
| #12 | (RNase 3):ti,ab,kw | 37 |
| #13 | (eosinophil major basic protein):ti,ab,kw | 24 |
| #14 | (major basic protein):ti,ab,kw | 407 |
| #15 | (eosinophil-derived neurotoxin):ti,ab,kw | 76 |
| #16 | (eosinophil derived neurotoxin):ti,ab,kw | 76 |
| #17 | (RNase 2):ti,ab,kw | 51 |
| #18 | (eosinophil peroxidase):ti,ab,kw | 65 |
| #19 | #9 OR #10 OR #12 OR #13 OR #14 OR #15 OR #16 OR #17 OR #18 | 855 |
| #20 | #8 AND #19 | 18 |

**Database:** Embase

**Final search date:** June 12, 2026

**Results retrieved:** 322

**Search strategy:**

('sinusitis'/exp OR 'nasal polyp'/exp OR 'chronic rhinosinusitis':ti,ab OR 'chronic sinusitis':ti,ab OR 'rhinosinusitis':ti,ab OR 'nasal polyp':ti,ab OR 'nasal polyposis':ti,ab) AND ('eosinophil granule protein'/exp OR 'eosinophil granule protein*':ti,ab OR 'eosinophil cationic protein':ti,ab OR 'rnase 3':ti,ab OR 'eosinophil major basic protein':ti,ab OR 'major basic protein':ti,ab OR 'eosinophil-derived neurotoxin':ti,ab OR 'eosinophil derived neurotoxin':ti,ab OR 'rnase 2':ti,ab OR 'eosinophil peroxidase':ti,ab)
